# Set-Theoretic Formalism for Treating Ligand-Target Datasets

**DOI:** 10.3390/molecules26247419

**Published:** 2021-12-07

**Authors:** Gerald Maggiora, Martin Vogt

**Affiliations:** 1 BIO5 Institute, University of Arizona, Tucson, AZ 85721, USA; 2 Department of Life Science Informatics, B-IT, LIMES Program Unit Chemical Biology and Medicinal Chemistry, Rheinische Friedrich-Wilhelms-Universität, Friedrich-Hirzebruch-Allee 5-6, D-53115 Bonn, Germany; martin.vogt@bit.uni-bonn.de

**Keywords:** polypharmacology, set theory, ternary relations, ligand-target interactions

## Abstract

Data on ligand–target (LT) interactions has played a growing role in drug research for several decades. Even though the amount of data has grown significantly in size and coverage during this period, most datasets remain difficult to analyze because of their extreme sparsity, as there is no activity data whatsoever for many LT pairs. Even within clusters of data there tends to be a lack of data completeness, making the analysis of LT datasets problematic. The current effort extends earlier works on the development of set-theoretic formalisms for treating thresholded LT datasets. Unlike many approaches that do not address pairs of unknown interaction, the current work specifically takes account of their presence in addition to that of active and inactive pairs. Because a given LT pair can be in any one of three states, the binary logic of classical set-theoretic methods does not strictly apply. The current work develops a formalism, based on ternary set-theoretic relations, for treating thresholded LT datasets. It also describes an extension of the concept of data completeness, which is typically applied to sets of ligands and targets, to the local data completeness of individual ligands and targets. The set-theoretic formalism is applied to the analysis of simple and joint polypharmacologies based on LT activity profiles, and it is shown that null pairs provide a means for determining bounds to these values. The methodology is applied to a dataset of protein kinase inhibitors as an illustration of the method. Although not dealt with here, work is currently underway on a more refined treatment of activity values that is based on increasing the number of activity classes.

## 1. Introduction

Data on ligand–target (LT) interactions has played a growing role in drug research over the last several decades. The amount of publicly available data has grown significantly in size and coverage during that period due to a number of factors including significant improvements in high-throughput assay methods and the greater availability of large, diverse compound libraries. Ligand–target datasets are a significant asset in many phases of drug discovery including high-throughput screening, lead identification and optimization, and as a basis for selecting compounds for pre-clinical development. Such datasets also provide information that can be used for the determination of pharmacological properties such as polypharmacologies (The terminology ‘polypharmacology’ does not explicitly distinguish polypharmacologies associated with single ligands from those associated with two or more ligands. Thus, the terminology *simple polypharmacology* and *joint polypharmacology* will be used when it is necessary to clearly distinguish between them. If there is no need to distinguish between them, the term polypharmacology, without any modifying adjective, will be employed.), which are of interest in a number of phases of drug research [1,2].

Ligand–target interaction data is typically handled in tables of some sort, where the rows nominally represent ligands and the columns represent pharmacologically relevant targets. Ideally, each cell in the table contains some measure of LT activity or interaction. As there are many different types of activity measures, obtaining a consistent measure can be difficult [3,4], especially in publicly available datasets, which tend to contain data from multiple sources. In some instances, computed quantitative interaction values are not given, only whether there is or is not some form of interaction. Because of this, it may be desirable to *threshold* known activity/interaction values, which is the approach taken in this work. Such a binary approach alleviates, to some extent, the problem that quantitative values from different sources and experimental protocols, e.g., binding affinities given as K_d_’s and inhibitory coefficients given as IC_50_′s, may not be directly comparable.

An important issue, noted by Mestres, et al. [5] more than a dozen years ago, is the substantial lack of data completeness in many LT datasets. This is a reflection of the fact that the activity, or lack thereof, of many LT pairs in such datasets have neither been experimentally nor computationally evaluated. As is discussed in this work, data completeness can be viewed in three ways: (1) as *global data completeness* (*GDC*), which is identical to that defined by Mestres, et al. [5]; (2) as *ligand-based local data completeness*, *LDC*(*l*), which is associated with the number of targets a given ligand is active, inactive, and null with respect to; and (3) as *target-based local data completeness, LDC*(*t*), which is associated with the number of ligands a given target is active, inactive, or null with respect to. Thus, measures of global data completeness afford less detailed information than that obtainable from measures associated with ligand-based or target-based local data completeness. As will be shown in Section 4, the average values of *LDC*(*l*) and *LDC*(*t*) are equal to one another and to the GDC.

Because publicly available datasets are usually comprised of data obtained from multiple sources, the data in these datasets tend to be of uneven quality, and to be quite sparse and inhomogeneously distributed, appearing much like ‘*Islands of data floating on a largely empty sea*’. In such instances, determining activity values computationally is a non-trivial task. Moreover, the expense of carrying out assays on such a massive scale, the lack of availability of some target proteins, and the difficulty associated with certain assays make it highly unlikely that large LT datasets will be fully populated with activity values in the near future. This raises a number of interesting questions that bear on the issue of how to represent information on the activity status of LT pairs in a way that not only takes account of active and inactive pairs but also accounts for the significant number of null pairs, i.e., pairs whose activity status is unknown. As is demonstrated below for polypharmacologies, null pairs provide a means for determining bounds to properties of interest in drug discovery research. An application of the formalism is exemplified by the determination of simple and joint polypharmacologies, as well as bounds to their values. This affords a means for determining polypharmacologies as interval values, and hence provides a measure of the uncertainty in their actual values. While this can lead to problems when comparing polypharmacologies for different ligands, they can be handled in most cases using the theory of semiorders [6]. It should also be noted that even large datasets do not include all biologically relevant ligands and targets, hence their estimates of polypharmacology and joint polypharmacology values are undoubtedly underestimates, although they are valid with respect to the ligand and target subsets being considered.

The mathematical framework used to treat LT datasets is typically based on some form of set theory. If the analysis is threshold-based, LT pairs whose activity/interaction value is equal to or exceeds a given threshold are considered to be ‘*active pairs’* and are assigned a value of unity. If they do not exceed the threshold, they are assigned a value of zero and are considered to be ‘*inactive pairs*’. A problem arises in the case of LT pairs for which neither experimentally nor computationally determined values exist. Such null pairs represent a degree of uncertainty with respect to the activity or inactivity of a given pair. In many instances they are neglected or given a value of zero, and thus are, de facto, included with the subset of inactives. Since a fraction of the null pairs would quite likely be active, assigning a zero value to them can result in a considerable undercounting of active pairs. As we shall see, this can have a significant impact on the bounds computed for simple and joint polypharmacologies. Moreover, as the number of null pairs can be quite large, even if only a small fraction of them are active, they could, nevertheless, contribute substantially to the number of active pairs. In addition, many null pairs would most likely be inactive if their activities were determined. As is discussed in Section 3.1 [See also Equation (40)], this can nevertheless have a significant effect on the bounds for polypharmacologies since every LT pair of known interaction, whether it corresponds to an active or inactive pair, reduces the number of null pairs, which play a significant role in determining bounds for polypharmacologies.

As the work here is data-driven, no attempt is made to address the lack of data completeness in most publicly available databases through the development of predictive computational methods. Rather, the aim is to develop a formalism that takes explicit account of the three activity states of LT pairs—active, inactive, and null. Because there are three states rather than the usual two in classical set theory, some extension of these methods is required. One could appeal to ternary rather than the usual binary logic [7], or to new forms of set theory such as intuitionistic fuzzy sets [8], both of which are likely to be unfamiliar to many researchers. We have chosen to develop an extension of classical set theory based on ternary relations. Although much less well known than their binary counterparts, ternary relations nevertheless fall within the purview of classical set theory [9]. An important feature of the method developed here is the use of *set-theoretic projections* to transform the initial, more complicated ternary relational form that is the basis of the method into the simpler and more familiar form of unary relations, which are identical to the one-dimensional sets usually described in classical set theories. Preliminary accounts of closely related work were recently published [10,11].

Section 2 describes the development of a set-theoretic formalism based on ternary relations that is capable of treating null LT pairs in addition to the usual active and inactive pairs. Section 2.1 deals with the basic set-theoretical preliminaries needed to complete this task. Section 2.2 presents an analysis of the equivalence classes associated with a number of the set-theoretic entities treated in this work. Section 2.3 describes the application of set-theoretic projections that decompose the original ternary relations into simpler unary relations (*a.k.a.* ‘traditional sets’) [12,13], which can then be used to compute various pharmaceutical properties. Section 3 describes an application of the method to the computation of polypharmacologies. Section 3.1 applies to simple polypharmacologies, while Section 3.2 deals with joint polypharmacologies. Although in almost all instances polypharmacologies are determined as point estimates, in this work they are also determined as interval values. This has important consequences for the potential magnitude of polypharmacologies, which this work shows may be severely underestimated. Section 4 presents an example of an application of the methodology developed here to a representative set of protein-kinase inhibitors. Section 5 summarizes the results and presents several conclusions on the relevance of the work to drug research.

## 2. Methods

### 2.1. Set-Theoretic Preliminaries

Classical ternary set-theoretic relations, ℛ(L,T,A), are given by

(1) ℛ(L,T,A)⊆L×T×A,

where L×T×A is the Cartesian product of the reference sets of ligands, *L*; of targets, *T*; and of activity states, *A*, given in Equations (2), (3) and (4). Respectively:

(2) $$L=\left\{l_{1},l_{2},\ldots ,l_{n}\right\},$$

(3) $$T=\left\{t_{1},t_{2},\ldots ,t_{m}\right\},$$

(4) $$A=\left\{a_{+},a_{-},a_{\emptyset }\;\right\}.$$

**Figure 1:**
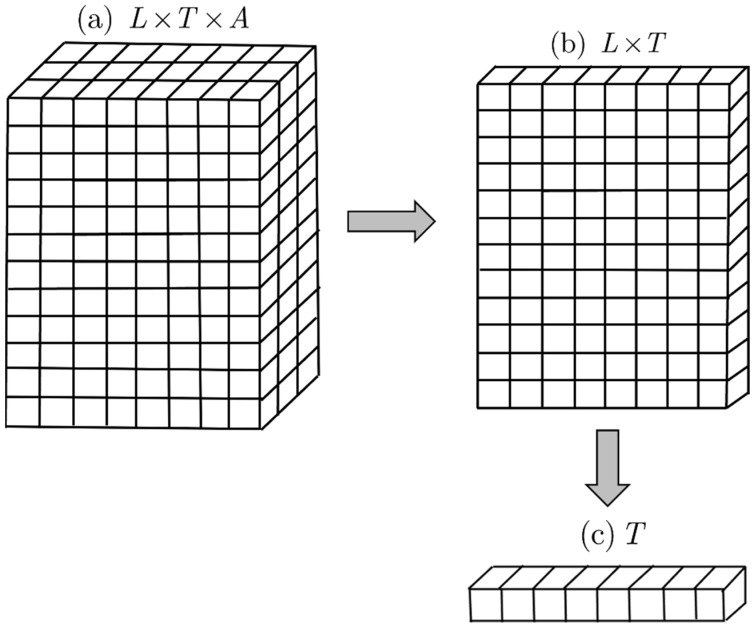
Simple examples of the (**a**) ternary, (**b**) binary, and (**c**) unary reference sets used in this work. The arrows correspond to set-theoretic projections from L×T×A⇒L×T and from L×T⇒T, respectively.

Thus, L×T×A is the reference set associated with the ternary relation and all of its subrelations. Because the reference set is three-dimensional, its elements are ordered triples, i.e., (l,t,a)∈L×T×A, where l∈L, t∈T, and a∈A. All set-theoretic operations such as unions, intersections, and cardinalities carried out on ℛ(L,T,A) and its subrelations must take place within this reference set. Figure 1a depicts a simple ternary reference set with 12 ligands, 8 targets, and 3 activity classes giving rise to a ternary reference set, which in this case has 12×8×3=288 elements. Figure 1b,c depict binary and unary reference sets obtained by set-theoretic projections of L×T×A and L×T, respectively. As lower-dimensional relations of the parent ternary relation will form the bulk of work described in this paper, binary and unary reference sets, especially the latter, will play important roles in this work.

**Figure 2:**
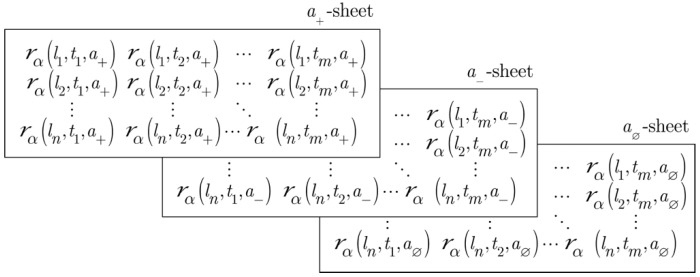
Three binary subrelations associated with ℛ(L,T,A), *viz.* ${ℛ}_{+}\left(L,T\right)$, ${ℛ}_{-}\left(L,T\right)$, and ${ℛ}_{\emptyset }\left(L,T\right)$, which correspond to the $a_{+}$-, $a_{-}$-, and $a_{\emptyset }$-sheet in the figure, respectively. They are formed by projections of the respective ternary subrelations ${ℛ}_{+}\left(L,T,A\right)$, ${ℛ}_{-}\left(L,T,A\right)$, and ${ℛ}_{\emptyset }\left(L,T,A\right)$ onto the binary reference set L×T.

As the reference set given in Equation (1) is three-dimensional, its associated relations and subrelations are also represented by three-dimensional matrices, which are difficult to portray in two dimensions. Figure 2 depicts an expanded version of the three-dimensional subrelations, ${ℛ}_{+}\left(L,T,A\right)$, ${ℛ}_{-}\left(L,T,A\right)$, and ${ℛ}_{\emptyset }\left(L,T,A\right)$, which are associated with active, inactive, or null LT pairs, respectively. Each rectangular sheet represents the set of LT pairs associated with a given element of a∈A, which are referred to as $a_{+}$-, $a_{-}$-, and $a_{\emptyset }$-sheets, respectively—they are offset from one another in order to clarify the relationship of all of the elements of the ternary relation to one another.

The characteristic functions of the ternary relation are

(5) $$r\left(l,t,a\right)=\{\begin{array}{cc} 1 & \mathrm{if}\;\left(l,t,a\right)\in ℛ\left(L,T,A\right)\\ 0 & \mathrm{otherwise} \end{array}$$

for all l∈L, t∈T, and, a∈A. Since a given LT pair must be either active, inactive, or null, and since their characteristic function values all lie in {0,1}, it follows from Equation (6) that if one of the terms has a value of unity, then the other two must have value of zero

(6) $$r\left(l,t,a_{+}\right)+r\left(l,t,a_{-}\right)+r\left(l,t,a_{\emptyset }\right)=1,$$

Equation (6) also shows that only two of its elements are independent.

The characteristic functions of the three subrelations ${ℛ}_{\alpha }\left(L,T,A\right)\subseteq ℛ\left(L,T,A\right)$, where α∈{+,−,∅}, are given for active pairs in Equation (7),

(7) $$r_{+}\left(l,t,a\right)=\{\begin{array}{ll} 1 & \mathrm{if}\;\left(l,t,a_{+}\right)\;\mathrm{is}\;\mathrm{an}\;\mathrm{active}\;\mathrm{pair}\\ 0 & \mathrm{if}\;\left(l,t,a_{+}\right)\;\mathrm{is}\;\mathrm{not}\;\mathrm{an}\;\mathrm{active}\;\mathrm{pair}\\ 0 & \mathrm{for}\;\mathrm{all}\;\left(l,t,a_{-}\right)\;\mathrm{and}\;\left(l,t,a_{\emptyset }\right) \end{array},$$

for inactive pairs in Equation (8),

(8) $$r_{-}\left(l,t,a\right)=\{\begin{array}{ll} 1 & \mathrm{if}\;\left(l,t,a_{-}\right)\;\mathrm{is}\;\mathrm{an}\;\mathrm{inactive}\;\mathrm{pair}\\ 0 & \mathrm{if}\;\left(l,t,a_{-}\right)\;\mathrm{is}\;\mathrm{not}\;\mathrm{an}\;\mathrm{inactive}\;\mathrm{pair}\\ 0 & \mathrm{for}\;\mathrm{all}\;\left(l,t,a_{+}\right)\;\mathrm{and}\;\left(l,t,a_{\emptyset }\right) \end{array},$$

and for null pairs in Equation (9),

(9) $$r_{\emptyset }\left(l,t,a\right)=\{\begin{array}{ll} 1 & \mathrm{if}\;\left(l,t,a_{\emptyset }\right)\;\mathrm{is}\;a\;\mathrm{null}\;\mathrm{pair}\\ 0 & \mathrm{if}\;\left(l,t,a_{\emptyset }\right)\;\mathrm{is}\;\mathrm{not}\;a\;\mathrm{null}\;\mathrm{pair}\\ 0 & \mathrm{for}\;\mathrm{all}\;\left(l,t,a_{+}\right)\;\mathrm{and}\;\left(l,t,a_{-}\right) \end{array}.\;$$

The relation and its subrelations satisfy

(10) $${ℛ}_{\alpha }\left(L,T,A\right)\subseteq ℛ\left(L,T,A\right)\subseteq L\times T\times A,$$

for α∈{+,−,∅}, and hence they all ‘live’ in the reference set L×T×A.

While some of the elements on all three of the ‘*a*-sheets’ in the parent relation ℛ(L,T,A) are likely to have non-zero values because most LT datasets have active, inactive, and null LT pairs, this is not necessarily the case with respect to all of its subrelations. Consider, for example, ${ℛ}_{+}\left(L,T,A\right)$. In this case, some of the elements (i.e., LT pairs) in the $a_{+}$-sheet belong to the set of actives; hence, $r_{+}\left(l,t,a_{+}\right)=1$, and some do not belong; hence, $r_{+}\left(l,t,a_{+}\right)=0$. However, the remaining elements in ${ℛ}_{+}\left(L,T,A\right)$ are zero, i.e., $r_{+}\left(l,t,a_{-}\right)=r_{+}\left(l,t,a_{\emptyset }\right)=0$ for all l∈L and t∈T. It is also important to note that while a value of $r_{+}\left(l,t,a_{+}\right)=1$ indicates that the designated LT pair is active, a value of $r_{+}\left(l,t,a_{+}\right)=0$ does not indicate that the pair is inactive, only that the $\left(l,t,a_{+}\right)$-th element does not belong to the subrelation ${ℛ}_{+}\left(L,T,A\right)$—it could belong to either of the other two subrelations. If the pair was known to be inactive it would belong in ${ℛ}_{-}\left(L,T,A\right)$, and if nothing were known about it, it would belong to ${ℛ}_{\emptyset }\left(L,T,A\right)$. Since all the subrelations satisfy Equation (10), their corresponding cardinalities satisfy

(11) $$|{ℛ}_{\alpha }\left(L,T,A\right)|\leq \left|ℛ\left(L,T,A\right)\right|\leq \left|L\times T\times A\right|=\left|L\right|\times \left|T\right|\times 3=n\times m\times 3,$$

where

(12) $$\left|ℛ\left(L,T,A\right)\right|=\sum_{l\in L}\sum_{t\in T}\sum_{a\in A}r\left(l,t,a\right)$$

and

(13) $$\left|{ℛ}_{\alpha }\left(L,T,A\right)\right|=\sum_{l\in L}\sum_{t\in T}\sum_{a\in A}r_{\alpha }\left(l,t,a\right),$$

where α∈{+,−,∅}.

### 2.2. Equivalence Classes of ℛ(L,T,A)

Consider the characteristic function values of the three subrelations, ${ℛ}_{\alpha }\left(L,T,A\right)$, that are summarized in Table 1. Each of the column headings refers to one of the rectangular sheets depicted in Figure 2. This clearly shows the partitioning of ℛ(L,T,A) since each sheet has non-zero values that are only associated with a single activity class: $a_{+}$-sheet ⟺ active, $a_{-}$-sheet ⟺ inactive, and $a_{\emptyset }$-sheet ⟺ null, i.e., only active LT pairs have non-zero values on the $a_{+}$-sheet, only inactive pairs have non-zero values on the $a_{-}$-sheet, and only null pairs have non-zero values on the $a_{\emptyset }$-sheet. Thus, the elements in each of the activity classes constitute an equivalence class.

Because the three subrelations partition the original ternary relation, their union gives the original ternary relation,

(14) $${ℛ}_{+}\left(L,T,A\right)\cup^{\;}{ℛ}_{-}\left(L,T,A\right)\cup^{\;}{ℛ}_{\emptyset }\left(L,T,A\right)=ℛ\left(L,T,A\right).$$

As the union operation is associative, and since a given element satisfies Equation (13), the elements of the union of the three subrelations satisfy

(15) $$r_{{ℛ}_{+}\cup^{\;}{ℛ}_{-}\cup^{\;}{ℛ}_{\emptyset }}\left(l,t,a\right)=\max\left[r_{+}\left(l,t,a\right),r_{-}\left(l,t,a\right),r_{\emptyset }\left(l,t,a\right)\right]\in \left\{0,1\right\}$$

for all l∈L,  t∈T, and a∈A. In addition, all three of the pairwise intersections of the subrelations are null,

(16) $$\begin{array}{c} {ℛ}_{+}\left(L,T,A\right)\cap^{\;}{ℛ}_{-}\left(L,T,A\right)={ℛ}_{+}\left(L,T,A\right)\cap^{\;}{ℛ}_{\emptyset }\left(L,T,A\right)=\\ {ℛ}_{-}\left(L,T,A\right)\cap^{\;}{ℛ}_{\emptyset }\left(L,T,A\right)=\emptyset , \end{array}$$

where the elements of the intersections are given by

(17) $$r_{{ℛ}_{\alpha }\cap^{\;}R_{\alpha^{\prime }}}\left(l,t,a\right)=\min\left[r_{\alpha }\left(l,t,a\right),r_{\alpha^{\prime }}\left(l,t,a\right)\right]=0$$

for all l∈L, t∈T, and a∈A, where $\alpha \neq \alpha^{\prime }$ and $\alpha ,\alpha^{\prime }\in \left\{+,-,\emptyset \right\}$. Thus, the cardinality of the ternary relation is equal to the sum of the cardinalities of the three subrelations (see Equations (12) and (13) for the mathematical expressions of their respective cardinalities),

(18) $$|ℛ\left(L,T,A\right)\left|=\right|{ℛ}_{+}\left(L,T,A\right)\left|+|{ℛ}_{-}\left(L,T,A\right)\right|+|{ℛ}_{\emptyset }\left(L,T,A\right)|.$$

### 2.3. Set-Theoretic Projections

Although all the manipulations needed to determine pharmaceutical properties such as polypharmacologies and target-based ligand similarities could be carried out in L×T×A, such an effort is quite inefficient because the majority of elements in these relations will have value zero. Fortunately, ternary and binary relations can be projected onto lower dimensional reference sets, which provide a much simpler basis for the computations [12] (pp. 122–124) and [13] (pp. 47–48).

**Figure 3:**
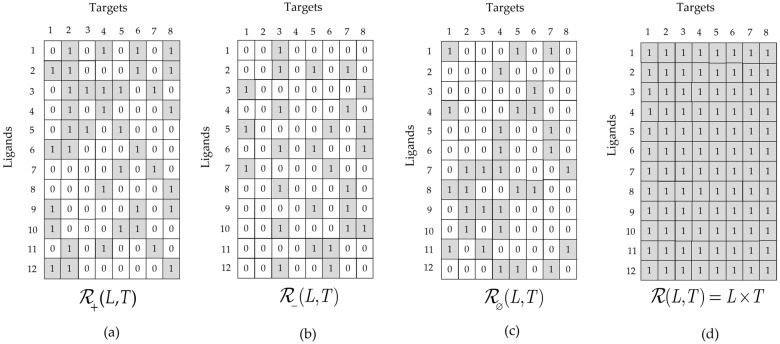
Example of three binary subrelations: (**a**) represents the set of active LT pairs, (**b**) represents the set of inactive LT pairs, (**c**) represents the set of null LT Pairs, and (**d**) represents their set-theoretic union, which is equivalent to the reference set L×T.

Consider the example given in Figure 3. Each of the three two-dimensional arrays depicted in Figure 3a–c represent projections (⇓)  of the subrelations in L×T×A onto the L×T reference set,

(19) $${ℛ}_{\alpha }\left(L,T\right)=\left[{ℛ}_{\alpha }\left(L,T,A\right)⇓L\times T\right],$$

where ${ℛ}_{\alpha }\left(L,T\right)$ is the projection of ${ℛ}_{\alpha }\left(L,T,A\right)$ onto L×T. As the projection is along the ‘*A*-axis’, there are three terms in ${ℛ}_{\alpha }\left(L,T,A\right)$ associated with each (l,t)-pair, $r_{\alpha }\left(l,t,a_{+}\right)$, $r_{\alpha }\left(l,t,a_{-}\right)$, and $r_{\alpha }\left(l,t,a_{\emptyset }\right)$. As shown in Equations (7)–(9), only one of the terms can be non-zero, i.e., $r_{\alpha }\left(l,t,a_{\alpha }\right)=1$ and $r_{\alpha }\left(l,t,a_{\alpha^{\prime }}\right)=r_{\alpha }\left(l,t,a_{\alpha^{\prime \prime }}\right)=0$, where $\alpha \neq \alpha^{\prime }\neq \alpha^{\prime \prime }$. Hence, the characteristic function obtained from the projection is given by

(20) $$r_{\alpha }^{⇓}\left(l,t,a\right)=\max\left[r_{\alpha }\left(l,t,a_{+}\right),r_{\alpha }\left(l,t,a_{-}\right),r_{\alpha }\left(l,t,a_{\emptyset }\right)\right]=r_{\alpha }\left(l,t\right)$$

for all l∈L and t∈T. This is equivalent to projecting the α-th sheet of ${ℛ}_{\alpha }\left(L,T,A\right)$ (see Figure 2) onto L×T because all of the elements in the other two sheets have a value of zero.

Because the original ternary subrelations, ${ℛ}_{\alpha }\left(L,T,A\right)$, are equivalence classes of ℛ(L,T,A), their projections onto L×T faithfully represent the information in their parent subrelations. Figure 3d shows the union of the three subrelations,

(21) $$ℛ\left(L,T\right)={ℛ}_{+}\left(L,T\right)\cup^{\;}{ℛ}_{-}\left(L,T\right)\cup^{\;}{ℛ}_{\emptyset }\left(L,T\right),$$

where in this special case ℛ(L,T)=L×T. As the ${ℛ}_{\alpha }\left(L,T\right)$ are equivalence classes, there is no overlap in their union. Thus,

(22) $$|ℛ\left(L,T\right)\left|=\right|{ℛ}_{+}\left(L,T\right)|+\left|{ℛ}_{-}\left(L,T\right)\right|+\left|{ℛ}_{\emptyset }\left(L,T\right)\right|=\left|L\times T\right|=n\times m.$$

The subrelation ${ℛ}_{\alpha ,l}\left(L,T\right)\subseteq {ℛ}_{\alpha }\left(L,T\right)$ associated with the *l*-th ligand is given by

(23) $${ℛ}_{\alpha ,l}\left(L,T\right)=\left(\begin{array}{cccc} 0 & 0 & \cdots & 0\\ \vdots & \vdots & \ddots & \vdots \\ 0 & 0 & \cdots & 0\\ r_{\alpha }\left(l,t_{1}\right) & r_{\alpha }\left(l,t_{2}\right) & \cdots & r_{\alpha }\left(l,t_{m}\right)\\ 0 & 0 & \cdots & 0\\ \vdots & \vdots & \ddots & \vdots \\ 0 & 0 & \cdots & 0 \end{array}\right),$$

where $r_{\alpha }\left(l^{\prime },t\right)=0$ for all $l^{\prime }\neq l$. It is clear from Equation (23) that ${ℛ}_{\alpha ,l}\left(L,T\right)$ can be projected onto the reference set *T*, i.e., $S_{\alpha }\left(l\right)=\left[{ℛ}_{\alpha ,l}\left(L,T\right)⇓T\right]$, whose elements are given by

(24) $$s_{\alpha }\left(l,t\right)=\max\left[r_{\alpha }\left(l_{1},t\right),r_{\alpha }\left(l_{2},t\right),\ldots ,r_{\alpha }\left(l_{n},t\right)\right]$$

for all t∈T, as all of the elements in each column of ${ℛ}_{\alpha ,l}\left(L,T\right)$ are zero except for the *l*-th element (some of which may also be zero), as shown in Equation (23). Hence, the activity information in ${ℛ}_{\alpha ,l}\left(L,T\right)$ is faithfully preserved in its projection,

(25) $$S_{\alpha }\left(l\right)=\left\{s_{\alpha }\left(l,t_{1}\right),s_{\alpha }\left(l,t_{2}\right),\ldots ,s_{\alpha }\left(l,t_{m}\right)\right\}\subseteq T$$

where α∈{+,−,∅}. The symbol $S_{\alpha }\left(l\right)$ is used here in order to emphasize the set-like character of these entities. (Equation (25) uses an alternative set notation by listing the characteristic function value of each element of the reference set. In the following, we will use this notation interchangeably with standard set notation).

Each $S_{+}\left(l\right)$ represents what could be called a *target-based ligand-activity profile*, or a *ligand-activity profile* (LAP) for short. In a similar vein, $S_{-}\left(l\right)$ and $S_{\emptyset }\left(l\right)$ could be referred to as *ligand-inactivity profiles* and *ligand-null profiles*, respectively. In some cases, they all will be referred to simply as ligand profiles. Each of the sets described by Equation (25) is a member of the family of related sets

(26) $$\{S_{\alpha }\}=\left\{S_{\alpha }\left(l_{1}\right),S_{\alpha }\left(l_{2}\right),\ldots ,S_{\alpha }\left(l_{n}\right)\right\}\subseteq T.$$

(As shown in Equation (26), all of the sets $S_{\alpha }\left(l\right)$ are subsets of the set of targets *T* since each one can be characterized as the subset of targets with a particular property, viz. that they are active with respect to a given ligand, *l.*),

Since the sets in Equation (26) are subsets of reference set *T*, all of the set-theoretic operations such as ‘intersection’ or ‘union’ can be performed on any pair of them. For example,

(27) $$S_{\alpha }\left(l\right)\cap^{\;}S_{\alpha^{\prime }}\left(l^{\prime }\right)=\left\{s_{\alpha }\left(l,t_{1}\right)\wedge s_{\alpha^{\prime }}\left(l^{\prime },t_{1}\right),\ldots ,s_{\alpha }\left(l,t_{m}\right)\wedge s_{\alpha^{\prime }}\left(l^{\prime },t_{m}\right)\right\},$$

and

(28) $$S_{\alpha }\left(l\right)\cup^{\;}S_{\alpha^{\prime }}\left(l^{\prime }\right)=\left\{s_{\alpha }\left(l,t_{1}\right)\vee s_{\alpha^{\prime }}\left(l^{\prime },t_{1}\right),\ldots ,s_{\alpha }\left(l,t_{m}\right)\vee s_{\alpha^{\prime }}\left(l^{\prime },t_{m}\right)\right\},$$

where ‘∧’ is the ‘min’ function, and ‘∨’ is the ‘max’ function.

Because of Equation (6), any given LT pair satisfies

(29) $$s_{+}\left(l,t\right)+s_{-}\left(l,t\right)+s_{\emptyset }\left(l,t\right)=1.$$

Therefore [*Cf.* Equations (14) and (16)],

(30) $$S_{+}\left(l\right)\cup^{\;}S_{-}\left(l\right)\cup^{\;}S_{\emptyset }\left(l\right)=T$$

and

(31) $$S_{+}\left(l\right)\cap^{\;}S_{-}\left(l\right)\cap^{\;}S_{\emptyset }\left(l\right)=\emptyset .$$

Hence, $S_{+}\left(l\right)$, $S_{-}\left(l\right)$, and $S_{\emptyset }\left(l\right)$ partition *T* for all l∈L, from which it follows that $S_{+}\left(l\right)$, $S_{-}\left(l\right)$, and $S_{\emptyset }\left(l\right)$ are equivalence classes. Thus, the elements (i.e., LT pairs) in $S_{+}\left(l\right)$ are all associated with active LT pairs, those in $S_{-}\left(l\right)$ are all associated with inactive pairs, and those in $S_{\emptyset }\left(l\right)$ are all associated with null pairs. While this does not affect the bounds on polypharmacologies, it does, as described in Section 3.2, significantly affect the bounds for joint polypharmacologies.

**Figure 4:**
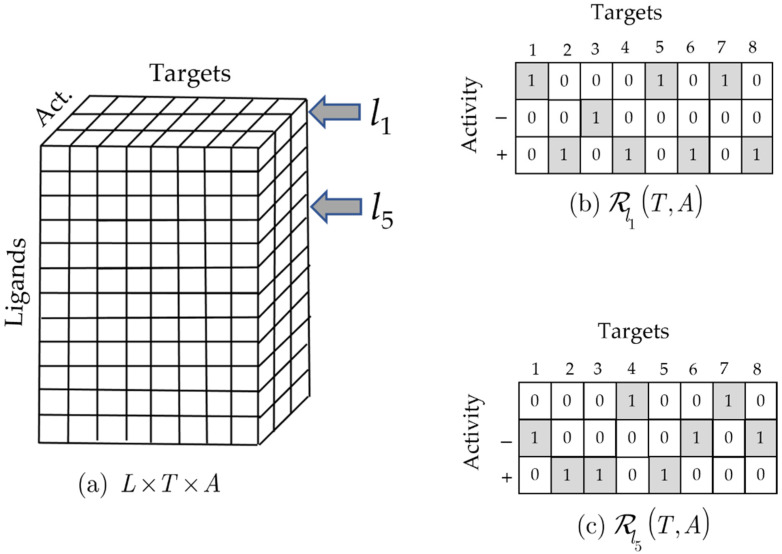
(**a**) Ternary reference set. (**b**) Ligand-based subrelation associated with $l_{1}$, ${ℛ}_{l_{1}}\left(T,A\right)\subseteq \left\{l_{1}\right\}\times T\times A$. (**c**) Ligand-based subrelation associated with $l_{5}$, ${ℛ}_{l_{5}}\left(T,A\right)\subseteq \left\{l_{5}\right\}\times T\times A$.

Because $S_{+}\left(l\right)$, $S_{-}\left(l\right)$, and $S_{\emptyset }\left(l\right)$ are equivalence classes that partition the set of targets, *T*,

(32) $$\left|S_{+}\left(l\right)\right|+\left|S_{-}\left(l\right)\right|+\left|S_{\emptyset }\left(l\right)\right|=\left|T\right|$$

and the corresponding fractions of active, inactive, and null LT pairs are given by

(33) $$f_{\alpha }\left(l\right)=\frac{\left|S_{\alpha }\left(l\right)\right|}{\left|T\right|},$$

where α∈{+,−,∅}, respectively. This is illustrated in Figure 4. The two arrows in Figure 4a point to two ‘slices’ associated with ligands $l_{1}$ and $l_{5}$ taken from the L×T×A reference set and depicted in Figure 4b,c. It is clear from these examples that Equations (29)–(31) are obtained for these two ligands, as is the case for all ligands treated by the formalism presented in this work.

## 3. Polypharmacologies

Although its existence is implied by the plethora of side effects associated with essentially all drugs, polypharmacology also plays an important, although not fully understood, role in determining the efficacy of many drugs. (Use of the terminology ‘polypharmacology’ implies that the interaction of a given ligand or drug with multiple biological targets induces multiple (i.e., ‘poly’) pharmacological responses. If a ligand/drug interacts in some fashion with multiple targets *without* inducing additional pharmacological responses, it would technically be called a promiscuous ligand or drug, but we will retain the polypharmacology nomenclature in this work.)Thus, polypharmacology, which is a manifestation of the seeming generality of ‘off-target’ interactions, raises a number of issues pertinent to drug research. The fact that a given ligand or drug can interact with multiple, largely protein, drug targets in the same functional class is not entirely unexpected [14]. The fact that in many instances it can also interact with multiple targets in different functional classes does, at least at first glance, seem surprising. Given the wide variety of surface topographies possessed by most targets, which are typically proteins, it is somewhat less surprising.

Studies aimed at identifying protein targets in different functional classes that interact with the same ligand have tended to focus on identifying structurally similar binding sites on these proteins. However, this is not an absolute requirement as shown by the recent, seminal work coming out of Shoichet’s laboratory [15], which showed that identical ligands can exhibit significantly different binding modalities in proteins in diverse functional classes. This finding provides a structural basis for the observed prevalence of ‘off-target’ interactions and hence, of polypharmacology.

An issue that arises in essentially all studies aimed at determining the values of polypharmacologies, is due to the fact that the datasets used to evaluate them are, to varying extents, incomplete, i.e., they do not contain activity values for all relevant LT pairs associated with a given ligand. The approach described in this work provides a scheme, albeit an incomplete one, for estimating bounds to polypharmacologies that may be able to provide at least some sense of the magnitudes of polypharmacologies (vide infra).

### 3.1. Simple Polypharmacologies

Simple polypharmacologies can be obtained by determining the cardinalities of appropriate LAPs, i.e.,

(34) $$P_{\min}\left(l\right)=\left|S_{+}\left(l\right)\right|=\sum_{t\in T}s_{+}\left(l,t\right),\;\mathrm{for}\;\mathrm{all}\;l\in L.$$

This would represent a lower bound to the true polypharmacology value since some of the null pairs would undoubtedly be active. Thus, a first approximation to an upper bound can be obtained by including null LT pairs and assuming they are all active. This is indicated by modifying the null pair index, $\emptyset \Rightarrow \emptyset^{+}$, so that

(35) $$P_{\max}\left(l\right)=\left|S_{+}\left(l\right)\right|+\left|S_{\emptyset^{+}}\left(l\right)\right|,$$

where

(36) $$\left|S_{\emptyset^{+}}\left(l\right)\right|=\sum_{t\in T}s_{\emptyset^{+}}\left(l,t\right)$$

for all l∈L. Note that the structures of $S_{\emptyset }\left(l\right)$ and $S_{\emptyset^{+}}\left(l\right)$ are identical, i.e., $S_{\emptyset }\left(l\right)\equiv S_{\emptyset^{+}}\left(l\right)\subseteq T$, although they are *interpreted* differently: the $s_{\emptyset }\left(l,t\right)=1$ terms represent LT pairs of unknown activity, while the $s_{\emptyset^{+}}\left(l,t\right)=1$ terms refer to LT pairs that are *assumed* to be active. The corresponding $s_{\emptyset }\left(l,t\right)=0$ and $s_{\emptyset^{+}}\left(l,t\right)=0$ terms represent LT pairs that do not belong to $S_{\emptyset }\left(l\right)$ and $S_{\emptyset^{+}}\left(l\right)$, respectively, but because of Equation (29) they must belong either to $S_{+}\left(l\right)$ or $S_{-}\left(l\right)$. The $s_{\emptyset^{+}}\left(l,t\right)$ terms will be used in the remainder of this section in order to emphasize that the null pairs are now assumed to represent active pairs. Thus, the ‘true’ value of the polypharmacology lies in the interval,

(37) $$P_{\min}\left(l\right)\leq P\left(l\right)\leq P_{\max}\left(l\right),$$

the size of which is equal to the maximum error (this, of course, is not the ‘true’ maximum error, since the *l*-th ligand may interact with many other targets not included within the specified target set.),

(38) $$P_{\max}\left(l\right)-P_{\min}\left(l\right)=\left|S_{\emptyset^{+}}\left(l\right)\right|=\Delta E_{\max}\left(l\right)$$

for all l∈L.

As noted in the Introduction, the number of null pairs in many instances is considerably larger than the number of active and inactive pairs combined. Thus, the upper bound in Equation (37) is generally too large to be of practical value. One approach is to reduce the overall size of the LT dataset by removing null pairs. Vogt, et al. [16] developed a novel procedure for removing them, but it can also remove some active pairs that could be of interest. By combining their method with that described here, it may be possible to obtain an optimal solution that removes a number of null pairs leading to a reduction in the error in a way that minimizes the loss of information due to the corresponding removal of active pairs. An alternative approach, as discussed below, involves estimation of the percentage of null pairs that are actually active.

The cardinality of the set of all LT pairs associated with the *l*-th ligand is given by

(39) $$\left|S_{+}\left(l\right)\right|+\left|S_{-}\left(l\right)\right|+\left|S_{\emptyset^{+}}\left(l\right)\right|=\left|T\right|,$$

Thus, the error can be written as

(40) $$\Delta E_{\max}\left(l\right)=\left|S_{\emptyset^{+}}\left(l\right)\right|=\left|T\right|-\left|S_{-}\left(l\right)\right|-\left|S_{+}\left(l\right)\right|,$$

for all l∈L. Obviously, since the size of *T* is fixed, $\left|S_{\emptyset^{+}}\left(l\right)\right|$ can be reduced by increasing the number of known inactives and actives, $\left|S_{-}\left(l\right)\right|$ and $\left|S_{+}\left(l\right)\right|$, respectively. An underappreciated issue in this regard is related to the number of inactive pairs, which tend to be underreported, especially if their activities are very low. This can materially affect the error, as is clear from Equation (40).

Since only a fraction of LT pairs are likely to be active, assuming that all null pairs in $S_{\emptyset^{+}}\left(l\right)$ are active is likely to be a substantial overestimate. Estimating the value of this fraction is, however, non-trivial as each of the LT pairs associated with that ligand involve a different pharmacological target. Hence, the ‘traditional’ approach would involve carrying out a set of QSAR-based (Quantitative Structure-Activity Relationships) computations, one for each target based on a structurally similar set of LT pairs of known activity associated with that target. Needless to say this is an arduous task and is unlikely to result in a balanced treatment of all relevant LT pairs. It is also a non-trivial task as most predictive methods, such as those associated with QSAR, employ linear models that generally are unsuitable for making predictions on structurally diverse sets of ligands and targets. While non-linear methods such as neural networks are better able to deal with this issue, they usually require substantial amounts of data in order to perform effectively [17,18,19]. Other approaches, including deep learning [20,21,22,23,24], have been examined, but the general sparseness and inhomogeneity of LT datasets tends to diminish the effectiveness of these types of approaches.

Under certain conditions, a simple procedure based on an ‘urn-like’ model may provide suitable estimates of the probability that a given LT pair associated with a specific ligand will be active [25]. Consider the fraction of actives of the *l*-th ligand with respect to the subset of targets for which activity data is available in the dataset. This is an estimate of the conditional probability that the null pairs associated with the *l*-th ligand are, in fact, active,

(41) $${\mathrm{Pr}}_{l}\left(\emptyset^{+}\right)\approx \frac{\left|S_{+}\left(l\right)\right|}{\left|S_{+}\left(l\right)+S_{-}\left(l\right)\right|}$$

for all l∈L. It provides an estimate of the probability that a randomly selected LT pair from $S_{\emptyset^{+}}\left(l\right)$ is active and, as shown in Equation (41), is based on the known actives and inactives associated with the *l*-th ligand. This estimate improves as the number of known active and inactive pairs associated with the ligand increases. In any case, it can only be considered to be a very approximate estimate, although it is considerably better than assuming that all LT pairs in $S_{\emptyset^{+}}\left(l\right)$ are active.

An ‘improved’ estimate of the number of active null pairs is given by

(42) $$\left|{\tilde{S}}_{\emptyset^{+}}\left(l\right)\right|={\mathrm{Pr}}_{l}\left(\emptyset^{+}\right)\cdot \left|S_{\emptyset^{+}}\left(l\right)\right|={\mathrm{Pr}}_{l}\left(\emptyset^{+}\right)\cdot \sum_{t\in T}s_{\emptyset^{+}}\left(l,t\right)\;\mathrm{for}\;\mathrm{all}\;l\in L.$$

Since probability is involved, $\left|{\tilde{S}}_{\emptyset^{+}}\left(l\right)\right|$ can be considered to be an expectation value. As mentioned above, inactive LT pairs are often underreported and thus the probability estimate of Equation (41) is overly optimistic and the estimate provided by Equation (42) is still likely to be an upper bound but not in a strict sense. An improved upper bound is given by

(43) $${\tilde{P}}_{\max}\left(l\right)=\left|S_{+}\left(l\right)\right|+\left|{\tilde{S}}_{\emptyset^{+}}\left(l\right)\right|\leq P_{\max},$$

where the ‘true’ value of P(l) lies in the interval given in Equation (44),

(44) $$P_{\min}\left(l\right)\leq P\left(l\right)\leq {\tilde{P}}_{\max}\left(l\right).$$

Hence, an improved error estimate is now given by

(45) $$\Delta \tilde{E}\left(l\right)=\left|{\tilde{S}}_{\emptyset^{+}}\left(l\right)\right|=\left[{\tilde{P}}_{\max}\left(l\right)-P_{\min}\left(l\right)\right]\leq \Delta E_{\max}\left(l\right).$$

### 3.2. Joint Polypharmacologies

Joint polypharmacologies are similar to simple polypharmacologies except that they are based on the number of targets that a pair of ligands are both active against. Paolini, et al. gave a closely related definition [26]. As will be seen in this section, the issue of joint targets significantly complicates estimates of upper bounds, so a more general approach is warranted to ensure that all appropriate terms are considered. This can be accomplished by constructing the augmented sets $S_{+}\left(l\right)\cup^{\;}S_{\emptyset }\left(l\right)$ and $S_{+}\left(l^{\prime }\right)\cup^{\;}S_{\emptyset }\left(l^{\prime }\right)$. Take their intersection and expand the expression using the distributive propety of classical sets [12]. Upon regrouping terms,

(46) $$\begin{array}{c} \left[S_{+}\left(l\right)\cup^{\;}S_{\emptyset }\left(l\right)\right]\cap^{\;}\left[S_{+}\left(l^{\prime }\right)\cup^{\;}S_{\emptyset }\left(l^{\prime }\right)\right]=\\ \left[S_{+}\left(l\right)\cap^{\;}S_{+}\left(l^{\prime }\right)\right]\cup^{\;}\left[S_{\emptyset }\left(l\right)\cap^{\;}S_{+}\left(l^{\prime }\right)\right]\cup^{\;}\left[S_{+}\left(l\right)\cap^{\;}S_{\emptyset }\left(l^{\prime }\right)\right]\cup^{\;}\left[S_{\emptyset }\left(l\right)\cap^{\;}S_{\emptyset }\left(l^{\prime }\right)\right]. \end{array}$$

In order to simplify the expressions, let

(47) $$\sum^{\;}\left(S\right)=\left[S_{+}\left(l\right)\cup^{\;}S_{\emptyset }\left(l\right)\right]\cap^{\;}\left[S_{+}\left(l^{\prime }\right)\cup^{\;}S_{\emptyset }\left(l^{\prime }\right)\right]$$

and

(48) $$S_{+,+}\left(l,l^{\prime }\right)=S_{+}\left(l\right)\cap^{\;}S_{+}\left(l^{\prime }\right)\;\mathrm{with}\;\left|S_{+,+}\left(l,l^{\prime }\right)\right|=\sum_{t\in T}s_{+}\left(l,t\right)\wedge s_{+}\left(l^{\prime },t\right),$$

(49) $$S_{+,\emptyset^{+}}\left(l,l^{\prime }\right)=S_{+}\left(l\right)\cap^{\;}S_{\emptyset^{+}}\left(l^{\prime }\right)\;\mathrm{with}\;\left|S_{+,\emptyset^{+}}\left(l,l^{\prime }\right)\right|=\sum_{t\in T}s_{+}\left(l,t\right)\wedge s_{\emptyset^{+}}\left(l^{\prime },t\right),$$

(50) $$S_{\emptyset^{+},+}\left(l,l^{\prime }\right)=S_{\emptyset^{+}}\left(l\right)\cap^{\;}S_{+}\left(l^{\prime }\right)\;\mathrm{with}\;\left|S_{\emptyset^{+},+}\left(l,l^{\prime }\right)\right|=\sum_{t\in T}s_{\emptyset^{+}}\left(l,t\right)\wedge s_{+}\left(l^{\prime },t\right),$$

(51) $$S_{\emptyset^{+},\emptyset^{+}}\left(l,l^{\prime }\right)=S_{\emptyset^{+}}\left(l\right)\cap^{\;}S_{\emptyset^{+}}\left(l^{\prime }\right)\;\mathrm{with}\;\left|S_{\emptyset^{+},\emptyset^{+}}\left(l,l^{\prime }\right)\right|=\sum_{t\in T}s_{\emptyset^{+}}\left(l,t\right)\wedge s_{\emptyset^{+}}\left(l^{\prime },t\right).$$

Thus, Equation (46) becomes

(52) $$\sum^{\;}\left(S\right)=S_{+,+}\left(l,l^{\prime }\right)\cup^{\;}S_{+,\emptyset^{+}}\left(l,l^{\prime }\right)\cup^{\;}S_{\emptyset^{+},+}\left(l,l^{\prime }\right)\cup^{\;}S_{\emptyset^{+},\emptyset^{+}}\left(l,l^{\prime }\right)$$

and their pairwise intersections are now given by

(53) $$\begin{array}{l} \emptyset =S_{+,+}\left(l,l^{\prime }\right)\cap^{\;}S_{+,\emptyset^{+}}\left(l,l^{\prime }\right)=S_{+,+}\left(l,l^{\prime }\right)\cap^{\;}S_{\emptyset^{+},+}\left(l,l^{\prime }\right)\\ \;=S_{+,+}\left(l,l^{\prime }\right)\cap^{\;}S_{\emptyset^{+},\emptyset^{+}}\left(l,l^{\prime }\right)=S_{+,\emptyset^{+}}\left(l,l^{\prime }\right)\cap^{\;}S_{\emptyset^{+},+}\left(l,l^{\prime }\right)\\ \;=S_{+,\emptyset^{+}}\left(l,l^{\prime }\right)\cap^{\;}S_{\emptyset^{+},\emptyset^{+}}\left(l,l^{\prime }\right)=S_{\emptyset^{+},+}\left(l,l^{\prime }\right)\cap^{\;}S_{\emptyset^{+},\emptyset^{+}}\left(l,l^{\prime }\right) \end{array}$$

which shows that $S_{+,+}\left(l,l^{\prime }\right),S_{+,\emptyset^{+}}\left(l,l^{\prime }\right),\;S_{\emptyset^{+},+}\left(l,l^{\prime }\right),\;\mathrm{and}\;S_{\emptyset^{+},\emptyset^{+}}\left(l,l^{\prime }\right)$ partition $\sum^{\;}\left(S\right)$, and hence, are equivalent classes. Thus,

(54) $$\left|\sum^{\;}\left(S\right)\right|=|S_{+,+}\left(l,l^{\prime }\right)\left|+\left|S_{+,\emptyset^{+}}\left(l,l^{\prime }\right)\right|+\left|S_{\emptyset^{+},+}\left(l,l^{\prime }\right)\right|+\right|S_{\emptyset^{+},\emptyset^{+}}\left(l,l^{\prime }\right)|$$

which is a general statement about the cardinality of $\sum^{\;}\left(S\right)$ given in Equation (47). It is clear that the situation in this instance is much more complex than that where the intersection of sets is not involved, as is the case for the simple polypharmacologies described in the previous section.

If it is assumed that the LT pairs in $S_{\emptyset }\left(l\right)$ and $S_{\emptyset }\left(l^{\prime }\right)$ are all inactive, then they effectively become null sets, i.e., $S_{\emptyset }\left(l\right)\Rightarrow \emptyset$ and $S_{\emptyset }\left(l^{\prime }\right)\Rightarrow \emptyset$. Hence, the summations in Equations (49)–(51) are equal to zero, and only the terms in Equation (48) are applicable. In such instances, the joint polypharmacology is a minimum, i.e.,

(55) $$P_{\min}\left(l,l^{\prime }\right)=\left|S_{+,+}\left(l,l\prime \right)\right|=\underset{t\in T}{\sum }s_{+}\left(l,t\right)\wedge s_{+}\left(l^{\prime },t\right).$$

If, as is the case for simple polypharmacologies, it is assumed that the elements of $S_{\emptyset }\left(l\right)$ and $S_{\emptyset }\left(l^{\prime }\right)$ correspond to active LT pairs, i.e., $S_{\emptyset }\left(l\right)\Rightarrow S_{\emptyset^{+}}\left(l\right)$ and $S_{\emptyset }\left(l^{\prime }\right)\Rightarrow S_{\emptyset^{+}}\left(l^{\prime }\right)$, the expressions in Equations (49)–(51) will likely contribute to the joint polypharmacology.

As was the case for polypharmacologies, assuming that all of the elements in these sets correspond to active LT pairs is likely to be a considerable overestimate, since the fraction of null LT pairs in many datasets is generally quite large. The maximum value of the joint polypharmacology involves all of the terms to the right of the equal sign in Equation (56),

(56) $$P_{\max}\left(l,l^{\prime }\right)=\left|S_{+,+}\left(l,l^{\prime }\right)\right|+\left|S_{+,\emptyset^{+}}\left(l,l^{\prime }\right)\right|+\left|S_{\emptyset^{+},+}\left(l,l^{\prime }\right)\right|+\left|S_{\emptyset^{+},\emptyset^{+}}\left(l,l^{\prime }\right)\right|,$$

so that the value of the joint polypharmacology lies in the interval

(57) $$P_{\min}\left(l,l^{\prime }\right)\leq P\left(l,l^{\prime }\right)\leq P_{\max}\left(l,l^{\prime }\right),$$

the size of which is equal to the maximum error

(58) $$\begin{array}{c} P_{\max}\left(l,l^{\prime }\right)-P_{\min}\left(l,l^{\prime }\right)=\left|S_{+,\emptyset^{+}}\left(l,l^{\prime }\right)\right|+\left|S_{\emptyset^{+},+}\left(l,l^{\prime }\right)\right|+\left|S_{\emptyset^{+},\emptyset^{+}}\left(l,l^{\prime }\right)\right|\\ =\Delta E_{\max}\left(l,l^{\prime }\right). \end{array}$$

As is the case with simple polypharmacologies, it is highly likely that only a fraction of the LT pairs in $S_{\emptyset^{+}}\left(l\right)$ and $S_{\emptyset^{+}}\left(l^{\prime }\right)$ are active. Thus a similar approach to that used in the case of polypharmacologies can also be applied here. This requires the following probability estimates:

(59) $${\mathrm{Pr}}_{l,l^{\prime }}\left(+,\emptyset^{+}\right)\approx \frac{\left|S_{+,+}\left(l,l^{\prime }\right)\right|}{\left|S_{+,+}\left(l,l^{\prime }\right)\right|+\left|S_{+,-}\left(l,l^{\prime }\right)\right|},$$

(60) $${\mathrm{Pr}}_{l,l^{\prime }}\left(\emptyset^{+},+\right)\approx \frac{\left|S_{+,+}\left(l,l^{\prime }\right)\right|}{\left|S_{+,+}\left(l,l^{\prime }\right)\right|+\left|S_{-,+}\left(l,l^{\prime }\right)\right|},$$

(61) $${\mathrm{Pr}}_{l,l^{\prime }}\left(\emptyset^{+},\emptyset^{+}\right)\approx \frac{\left|S_{+,+}\left(l,l^{\prime }\right)\right|}{\left|S_{+,+}\left(l,l^{\prime }\right)\right|+\left|S_{+,-}\left(l,l^{\prime }\right)\right|+\left|S_{-,+}\left(l,l^{\prime }\right)\right|+\left|S_{-,-}\left(l,l^{\prime }\right)\right|},$$

which are similar in general form to that given in Equation (41) for polypharmacologies.

These relationships provide a means for improving the upper bounds in a similar fashion to that employed for simple polypharmacologies in Section 3.1. Thus,

(62) $$\begin{array}{c} {\tilde{P}}_{\max}\left(l,l^{\prime }\right)=\left|S_{+,+}\left(l,l^{\prime }\right)\right|+{\mathrm{Pr}}_{l,l^{\prime }}\left(+,\emptyset^{+}\right)\cdot \left|S_{+,\emptyset^{+}}\left(l,l^{\prime }\right)\right|+{\mathrm{Pr}}_{l,l^{\prime }}\left(\emptyset^{+},+\right)\\ \cdot \;\left|S_{\emptyset^{+},+}\left(l,l^{\prime }\right)\right|+{\mathrm{Pr}}_{l,l^{\prime }}\left(\emptyset^{+},\emptyset^{+}\right)\cdot \;\left|S_{\emptyset^{+},\emptyset^{+}}\left(l,l^{\prime }\right)\right|, \end{array}$$

which simplifies to

(63) $${\tilde{P}}_{\max}\left(l,l^{\prime }\right)=\left|S_{+,+}\left(l,l^{\prime }\right)\right|+\left|{\tilde{S}}_{+,\emptyset^{+}}\left(l,l^{\prime }\right)\right|+\left|{\tilde{S}}_{\emptyset^{+},+}\left(l,l^{\prime }\right)\right|+\;\left|{\tilde{S}}_{\emptyset^{+},\emptyset^{+}}\left(l,l^{\prime }\right)\right|.$$

As before, the terms with tildes (‘~’) should be interpreted as expectation values, and, as is the case for simple polypharmacologies, the ‘true’ value of a joint polypharmacology lies in the interval

(64) $$P_{\min}\left(l,l^{\prime }\right)\leq P\left(l,l^{\prime }\right)\leq {\tilde{P}}_{\max}\left(l,l^{\prime }\right).$$

The size of the interval is bounded from below and above by the relevant error terms,

(65) $$\begin{array}{c} \Delta \tilde{E}\left(l,l^{\prime }\right)=\left[{\tilde{P}}_{\max}\left(l,l^{\prime }\right)-P_{\min}\left(l,l^{\prime }\right)\right]=\left|{\tilde{S}}_{+,\emptyset^{+}}\left(l,l^{\prime }\right)\right|\\ +\left|{\tilde{S}}_{\emptyset^{+},+}\left(l,l^{\prime }\right)\right|+\left|{\tilde{S}}_{\emptyset^{+},\emptyset^{+}}\left(l,l^{\prime }\right)\right|\leq \Delta E_{\max}\left(l,l^{\prime }\right), \end{array}$$

which are similar in form, albeit more complex, than the corresponding terms for polypharmacologies given in Equation (45).

## 4. Results and Discussion

An example, based on a protein kinase dataset (Supplementary Materials), is presented that illustrates the methodology described in this paper. Protein kinases represent an important target class with more than 85 clinically approved drugs to date. Moreover, research on new kinase inhibitors occupies about 30% of current R&D spending within the pharmaceutical industry [27]. While early studies were primarily focused on cancer chemotherapies [28], more recent studies have been expanded to include inflammatory processes as well [29,30,31].

To illustrate an application of the method, a dataset containing 250 ligands and a representative set of 433 protein kinases was obtained from ChEMBL version 28 [32]. It consists of data from two panel assays carried out by Klaeger, et al. [33] on 241 drugs and drug candidates and 319 kinases, and by Karaman et al. [34] on 38 ligands and 282 kinases. These data sets were augmented with a small amount of additional data from ChEMBL. The activity status of an LT pair was considered to be inactive if it was explicitly annotated as ‘not active’ or when the K_d_ or K_i_ value was larger than 3 μM (corresponding to a pK_d_ or pK_i_ of 5.5). In the case of conflicting activity annotations, the activity status was set to ‘null’. Table 2 provides a summary of the statistics of the PK dataset. The activity annotations have been made available as supplementary material. This work is focused on an analysis of the activity status of LT pairs in the dataset: it does not attempt to compute missing activity values or to address any structural issues associated with the ligands or the protein-kinase target proteins.

**Figure 5:**
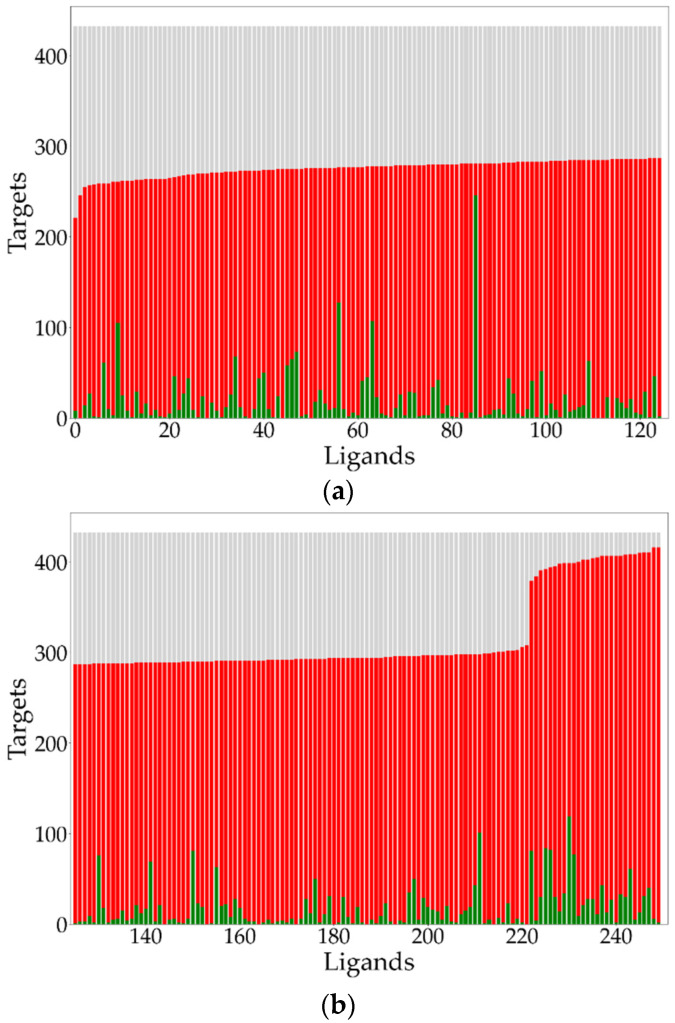
(**a**) Ligand activity profiles for the first 125 ligands out of the set of 250 ligands. (**b**) Ligand activity profiles for the remaining 125 ligands out of the set of 250 ligands. For each ligand, the number of active, inactive, and null targets are shown as green, red, and grey bars, respectively.

The activity profile of all 250 ligands is depicted in Figure 5a,b. Each ligand has three components that contribute to its overall activity profile, whose values are based on ‘counts’ of targets measured along the ordinate: (1) the count of targets that exhibit activities equal to or greater than the threshold value (depicted by the green bar), (2) the count of targets that exhibit activities that are less than the threshold value (depicted by the red bar), and (3) the count of targets for which there is no activity information (depicted by the grey bar). Ligands are ordered along the abscissa in increasing value of the count of active plus inactive LT pairs. As expected, in most instances, the number of inactive pairs for each ligand is significantly larger than the number of active pairs. The large area of grey clearly shows that in most cases there is a substantial lack of global data completeness in this dataset [5].

The ratio of the number of active plus inactive targets (green plus red bars) for the *l*-th ligand divided by the number of targets (green plus red plus grey bars), represents the ligand-based LDC with respect to the *l*-th ligand, i.e.,

(66) $$LDC\left(l\right)=\frac{\left|S_{+}\left(l\right)\right|+\left|S_{-}\left(l\right)\right|}{\left|T\right|}.$$

where $\left|T\right|=\left|S_{+}\left(l\right)\right|+\left|S_{-}\left(l\right)\right|+\left|S_{\emptyset }\left(l\right)\right|$. The average value of LDC(l) is given by

(67) $$\begin{array}{c} <LDC{(l)>}_{ave}=\frac{1}{\left|L\right|}\underset{l\in L}{\sum }LDC\left(l\right)=\frac{1}{\left|L\right|}\underset{l\in L}{\sum }\frac{\left|S_{+}\left(l\right)\right|+\left|S_{-}\left(l\right)\right|}{\left|T\right|}\\ =\frac{\sum_{l\in L}\left|S_{+}\left(l\right)\right|+\left|S_{-}\left(l\right)\right|}{\left|L\right|\left|T\right|}=GDC. \end{array}$$

**Figure 6:**
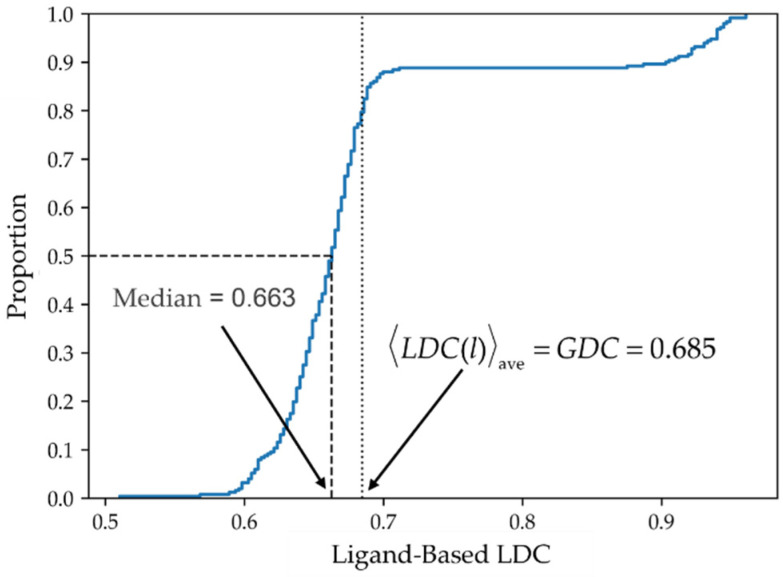
Cumulative distribution of ligand-based local data completeness, LDC(l). The cumulative distribution of the ligand data completeness for all 250 ligands is shown in blue.

Figure 6 depicts the cumulative distribution of LDC(l) values (Note that italics are used to indicate functions; e.g. *LDC*(*l*) and *LDC*(*t*)). As seen in the figure, the median and average values are of comparable magnitude, a condition that typically corresponds to a reasonably symmetric probability distribution.

**Figure 7:**
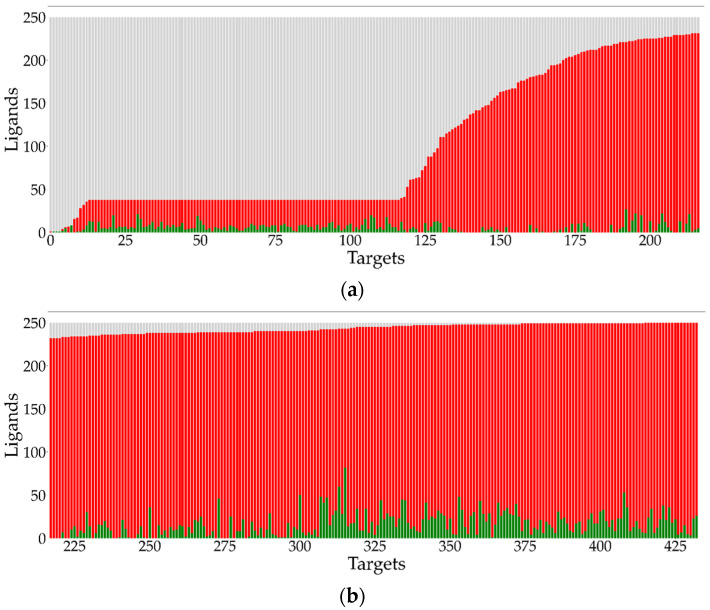
(**a**) Target activity profiles for the first 220 targets out of the set of 433 protein kinase targets. (**b**) Target activity profiles for the remaining 213 targets out of the set of 433 protein kinase targets. For each target, the number of active, inactive, and null ligands are shown as green, red, and grey bars, respectively.

**Figure 8:**
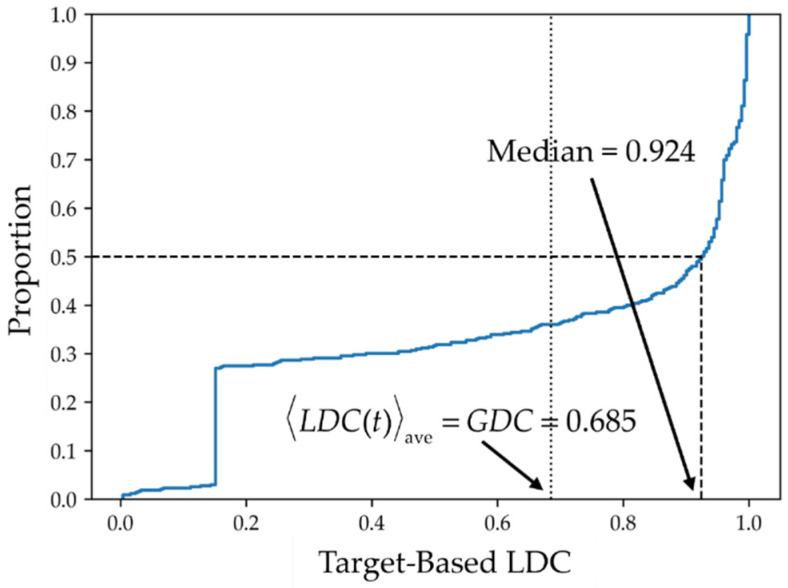
Cumulative distribution of target-based local data completeness, LDC(t). The cumulative distribution of the target data completeness of all 433 ligands is shown.

The same analysis can be carried out for target-based LDC’s, which are displayed in Figure 7. In each of the panels, ligands are indicated along the ordinates and targets along the abscissas. It is clear from these diagrams that the distribution of target-based LDC’s differs considerably from those of the ligand-based LDC’s depicted in Figure 5. A similar analysis to that given in Equation (67) yields an analogous result $<LDC{(t)>}_{ave}=GDC$ for target-based LDC’s, which shows that all three entities are equal to one another, i.e., $<LDC{(t)>}_{ave}=GDC=<LDC{(l)>}_{ave}$. Figure 8 depicts the cumulative distribution of target-based LDC values that, not surprisingly, show that the distributions are quite different, although as noted above, their average values are identical.

**Figure 9:**
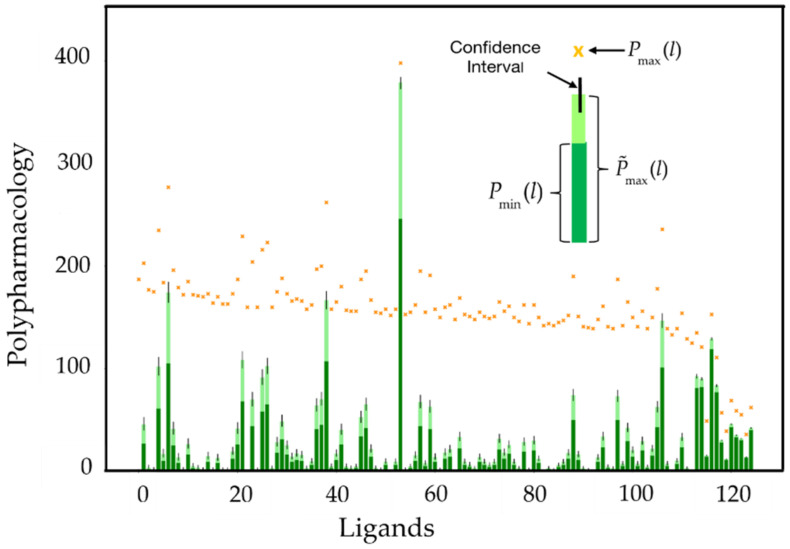
Histogram of 125 estimated polypharmacology values randomly sampled from the set of 250 ligands. The dark green bar represents the minimum polypharmacology, while the orange marks represent the maximum polypharmacology. The probabilistically estimated maximum polypharmacology is indicated by the composite dark and lime green bars and includes a 95% confidence interval indicated by the vertical black line.

A random sample of 125 ligands was selected from the original dataset of 250 ligands as a basis for illustrating the computation of polypharmacologies. Figure 9 depicts a histogram of 125 simple polypharmacology values. The dark green bars correspond to the minimum value of the polypharmacology, $P_{\min},$ given in Equation (34). The dark plus light green composite bars represent an estimate of the upper bound to the true polypharmacology value, based on Equations (41)–(43). As shown by these equations, the approximation depends on an estimate of the probability, given in Equation (41), that a null pair is actually active. This can be modelled as the number of successes in repeated Bernoulli trials, which yield a binomial distribution [35], from which confidence intervals at a significance level of α=0.05, represented by the vertical black lines in Figure 9, have been determined based on ‘Jeffreys Bayesian intervals’ [36].

**Figure 10:**
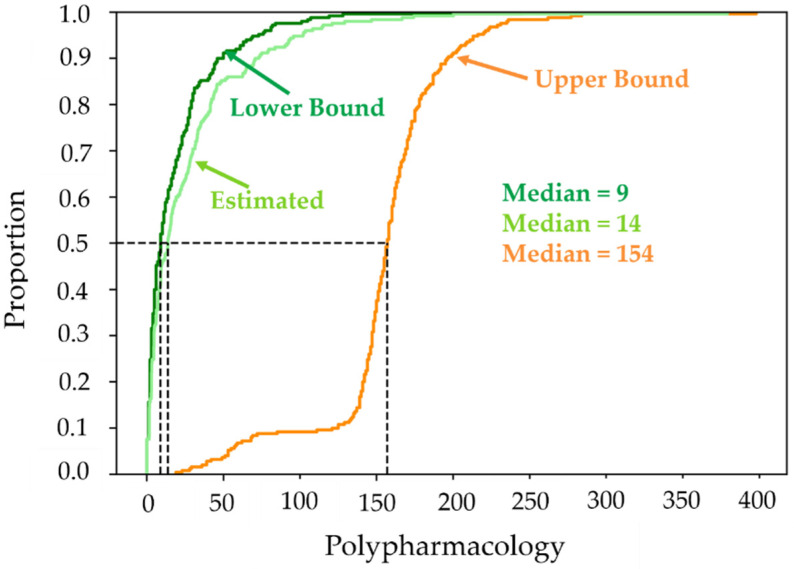
The cumulative distribution of the minimum, maximum, and estimated simple polypharmacologies for all 250 ligands are displayed in dark green, orange, and lime green, respectively.

Cumulative distributions of the simple polypharmacologies are given in Figure 10. The lower bounds, $P_{\min},$ are indicated by the dark green curve; the probabilistically estimated upper bounds, ${\tilde{P}}_{\max},$ are indicated by the lime-green curve; and the upper bounds, $P_{\max},$ are indicated by the orange curve. It is clear from the figure that the estimated values, which are based on Equations (42) and (43), lie between the max and min values, but lie close to the cumulative distribution of the minimum values. This is obviously a better approximation of the upper bound than that afforded by $P_{\max},$ which Figure 10 shows is a considerable overestimate of the true value of the simple polypharmacology. The minimum and estimated values also show that the magnitudes of simple polypharmacology are significant, with nearly 50% of the ligands exhibiting values of approximately 10 or greater. Part of the issue here is undoubtedly due to the fact that all of the targets are protein kinases, and hence come from a single activity class whose binding-site structures are well known to be somewhat structurally similar. Nevertheless, the high values of simple polypharmacologies are larger than is generally assumed. This can certainly pose problems for drug research, especially with regard to the development of drugs that are highly specific for a limited subset of protein-kinase targets. It does not address issues associated with non-protein-kinase targets. Hence, the ligands examined here could also exhibit significant levels of polypharmacology with respect to non-protein kinase targets as well.

**Figure 11:**
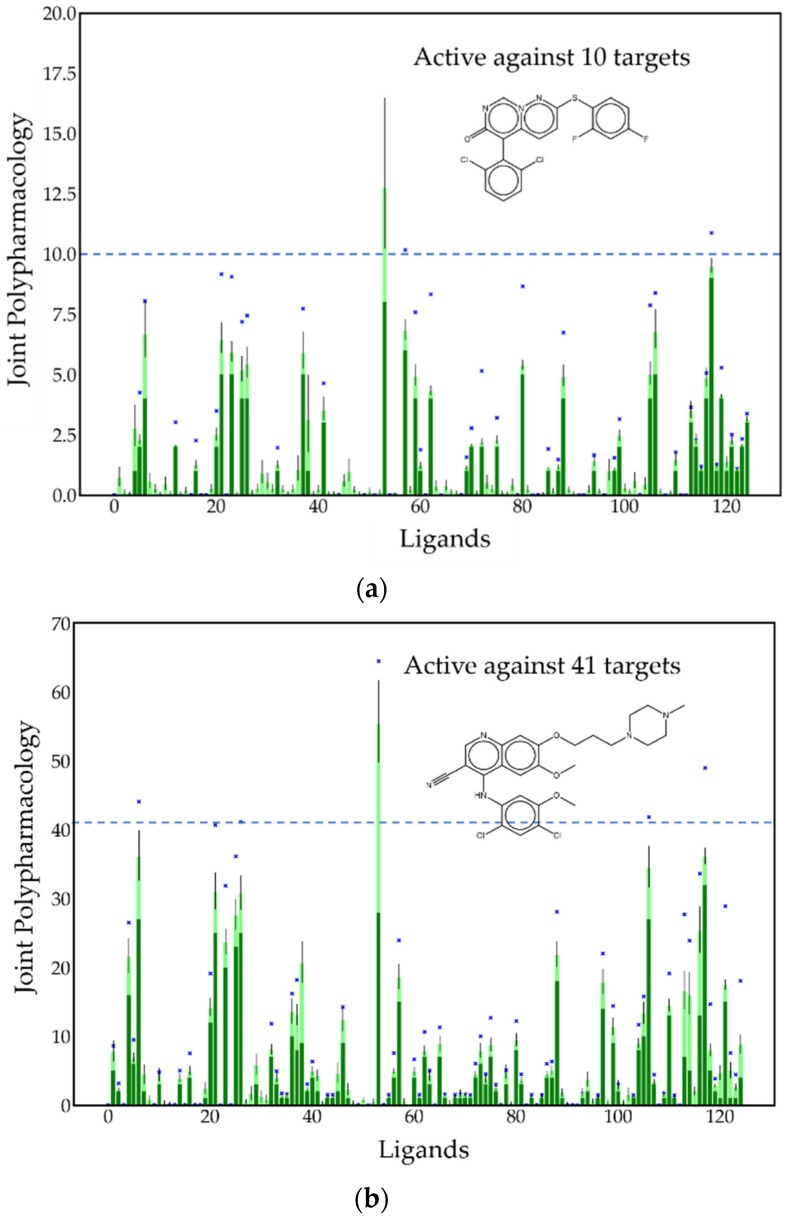
Histogram of estimated joint polypharmacology values for two exemplary ligands. (**a**) CHEMBL119385 and (**b**) CHEMBL288441 taken from the set of 125 ligands of Figure 9 are shown with respect to the other 124 exemplary ligands. The bars represent estimates based on assuming that probabilities are only dependent on the activities of individual ligands, i.e., ${\mathrm{Pr}}_{l,l^{\prime }}\left(\emptyset^{+},\emptyset^{+}\right)={\mathrm{Pr}}_{l}\left(\emptyset^{+}\right){\mathrm{Pr}}_{l^{\prime }}\left(\emptyset^{+}\right)$. The dark green bars represent the minimum joint polypharmacologies, and the lime green bars represent the contributions from LT pairs where one or both of them are null. The blue x’s show the estimates based on the joint activities of non-null LT pairs according to Equation (63).

The situation in the case of joint polypharmacologies is far more complicated since it involves set intersections over a set of n(n−1)/2  unique pairs of ligands. For the full set of n=250 ligands, this amounts to 31,125 pairs. Even for the reduced set of n=125 randomly sampled ligands, this still amounts to 7750 pairs. In order to reduce the amount of data and still provide a meaningful illustration of the methodology, two ligands, one which is active with respect to 10 targets and the other which is active against 41 targets, were chosen from the set of 125 randomly sampled ligands treated earlier, and their interaction with the other 124 ligands in the randomly sampled set was determined. This yielded two sets of 124 data points depicted in Figure 11a,b.

**Figure 12:**
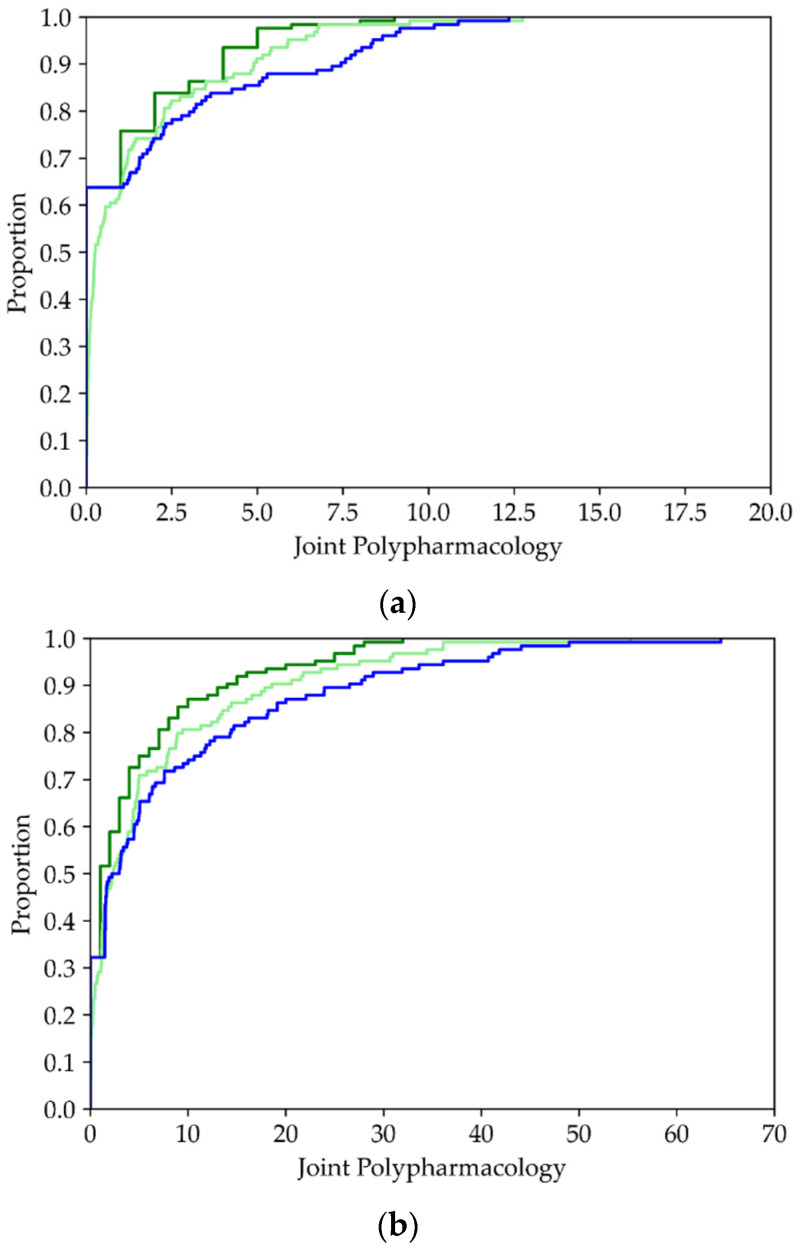
Cumulative distributions of estimated joint polypharmacologies for the exemplary ligands (**a**) CHEMBL119385 and (**b**) CHEMBL288441. The dark green line shows the cumulative distribution for the minimum joint polypharmacologies, the lime green for the estimated joint polypharmacologies assuming independence of activity for both ligands, and the blue line represents the estimate based on the observed joint polypharmacologies for non-null LT pairs.

The dark green bars in Figure 11 correspond to minimum joint polypharmacology values, which are calculated using the expression given in Equation (55). The probabilistically estimated upper bounds given by Equations (62) and (63) are represented by the composite dark and light green bars associated with each ligand. The probability estimates used in these equations are based on the assumption that the variables are independent, in which case ${\mathrm{Pr}}_{l,l^{\prime }}\left(\emptyset^{+},\emptyset^{+}\right)={\mathrm{Pr}}_{l}\left(\emptyset^{+}\right){\mathrm{Pr}}_{l^{\prime }}\left(\emptyset^{+}\right)$. Probability estimates taking account of dependencies between the variables, i.e., accounting for the notion that similar ligands might have similar activity profiles, are given in Equations (59)–(61). Applying these estimates to the expression in Equation (62) yields an estimate of the upper bound to the joint polypharmacology that is depicted as a single blue x for each ligand. Interestingly, this estimate of ${\tilde{P}}_{\max}$ is in essentially all cases significantly greater than the value obtained assuming statistical independence. Moreover, it also lies considerably above the confidence interval. This strongly suggests that the joint probabilities are consistently larger than those computed using the independence assumption. Thus, the corresponding joint polypharmacologies would be expected to exhibit similar behavior as is observed in Figure 11, and by the cumulative distributions depicted in Figure 12, which afford direct confirmation of this observation. As was the case for simple polypharmacologies, the black vertical lines correspond to confidence intervals that in this case are associated with joint polypharmacologies.

Because joint polypharmacologies involve set intersections, which satisfy $\left|S_{+}\left(l\right)\cap^{\;}S_{+}\left(l^{\prime }\right)\right|\leq \min\left[\left|S_{+}\left(l\right)\left|,\right|S_{+}\left(l^{\prime }\right)\right|\right]$, it follows that $P_{\min}\left(l,l^{\prime }\right)\leq \min\left[P_{\min}\left(l\right),P_{\min}\left(l^{\prime }\right)\right]$, which determines an upper bound to the minimum value of the joint polypharmacology. This is seen in Figure 11a,b, both of which depict this type of behavior quite clearly: (1) the molecule depicted in Figure 11a is active with respect to 10 targets, thus the maximum value of the minimum joint polypharmacologies associated with this ligand must satisfy $P_{\min}\left(l,l^{\prime }\right)\leq P_{\min}\left(l\right)=10$, and (2) the molecule depicted in Figure 11b is active with respect to 41 targets, thus the maximum value of the minimum joint polypharmacologies associated with this ligand must satisfy $P_{\min}\left(l,l^{\prime }\right)\leq P_{\min}\left(l\right)=41$. Both of these restrictions are clearly seen to apply in Figure 11a,b; in particular, note the horizontal dashed lines in both figures. In any case, the magnitude of many of the joint polypharmacologies is still quite significant. Hence, all of this data strongly suggests that polypharmacologies are likely to play a significant role in drug discovery that goes well beyond their role as inducers of side effects.

## 5. Summary and Conclusions

Almost all LT datasets exhibit a significant lack of data completeness as reflected by the substantial fraction of their LT pairs for which activity data is absent. Thus, there are three activity states—active, inactive, and unknown or null—rather than the two-state description (i.e., active and inactive) usually applied to these datasets. The two-state approach ignores a significant portion of the dataset, which is either neglected or is lumped together with the subset of inactive pairs. As some of the null pairs are undoubtedly active, this can lead to a significant undercounting of simple and joint polypharmacologies.

The current work develops a classical set-theoretic procedure based on ternary relations for representing activity-thresholded LT datasets composed of active, inactive, and null LT pairs. These relations are further simplified by projecting them onto simpler one-dimensional sets, facilitating the analysis of these datasets. Two applications of this methodology are described. The first involves a generalization based on the data completeness associated with individual ligands and targets, which affords a more highly resolved representation than the global view adopted in its original description [5]. The second involves the estimation of upper bounds to simple and joint polypharmacologies that are based on the subsets of null LT pairs. These values also provide the means for obtaining interval values for polypharmacologies. Both procedures are illustrated by an analysis of a protein-kinase dataset obtained from the ChEMBL database. It should be emphasized that the procedures developed here are aimed strictly at an analysis of the data; they are not meant to be QSAR tools for predicting the activities of individual molecules.

Although they do not have the size and complexity of LT datasets within the pharmaceutical industry, publicly available datasets are becoming more readily available with a greater diversity of content. However, because they are usually assembled from multiple datasets, they tend not to be uniform in coverage and reliability and exhibit a significant lack of data completeness (vide supra). This is unlikely to change significantly in the near future. Thus, the current approach affords a means for explicitly accounting for LT pairs of unknown activity that, although somewhat limited, may nevertheless provide information on pharmacological parameters such as ligand- and target-based LDC and on simple and joint polypharmacologies, which should be of interest in drug research. The present work does not fully address the issue of missing data per se. It does, however, describe a means for accounting for missing data, which is illustrated by the computation of *interval values* for simple and joint polypharmacologies, two key pharmacological parameters that have gained in importance in drug research.

Although the work presented here deals with thresholded datasets, an extension to datasets whose elements are taken from concentration-dependent activity data can be developed by increasing the degree of partitioning of the datasets. Thus, instead of partitioning LT pairs with known activity values into active and inactive subsets based on a single, assumed activity threshold, they can be more highly partitioned by defining several activity thresholds, which will define the boundaries of a number of partitions. Work is currently underway for the development of this modification that, not surprisingly, is considerably more complex than the method presented in this work. An interesting feature of the new work is the possibility of defining activity-dependent polypharmacologies—sets of LT pairs associated with low activities would give rise to ‘weak polypharmacologies’, while sets of LT pairs associated with high activities would give rise to ‘strong polypharmacologies’, and so on. The present approach can also be used to address target-based similarities, but because similarities involve ratios, and because their analysis must account for terms associated with null pairs, this adds an additional level of complication to the analysis, and thus their development will be dealt with at a later date.

## Figures and Tables

**Table 1:** Summary of characteristic function values for the three subrelations.

	$a_{+}$-Sheet	$a_{-}$-Sheet	$a_{\emptyset }$-Sheet
Active	{0,1}	0	0
Inactive	0	{0,1}	0
Null	0	0	{0,1}

**Table 2:** Global data completeness of PK dataset [5].

No. of Ligands	No. of Targets	Active LT Pairs	Inactive LT Pairs	Null LT Pairs	Total LT Pairs	Global Data Completeness
250	433	4,729	69,419	34,104	108,250	0.685

## Data Availability

The kinase activity annotations extracted from ChEMBL are available as supplementary material.

## Supplementary Materials

The following are available online. The activity annotations extracted from ChEMBL version 28 for 433 kinases and 250 ligands including target and ligand specifications are available as supplementary CSV files.
